# Infections in patients with adverse reactions to the use of unknown modeling substances for soft tissue enhancement in Cali, Colombia

**DOI:** 10.1371/journal.pone.0277958

**Published:** 2023-02-09

**Authors:** Jennifer Bonilla Moncada, Carlos Alberto Ríos, Claudia Marcela Castro, Aura Lucia Leal, Jhann Andres Arturo, Katty Diaz, Carolina Duarte, Gloria Puerto, Nancy Moreno, Amelia Velasco, Jaime Moreno

**Affiliations:** 1 Department of Microbiology, National Health Institute, Bogotá, Colombia; 2 Biotechnology Institute, National University of Colombia, Bogotá, Colombia; 3 Santuario Medical Center, Cali, Colombia; 4 Department of Mycobacteria, National Health Institute, Bogotá, Colombia; 5 Inmugen Corporation, Bogotá, Colombia; 6 INVIMA, Bogotá, Colombia; Babasaheb Bhimrao Ambedkar University, INDIA

## Abstract

The infiltration of foreign materials not approved for medical purposes or of modeling substances used in soft tissue to modify the anatomical appearance for aesthetic purposes represents a serious health problem. These procedures lead to the development of delayed complications, including infections. The objective of this study was to characterize infections in patients with adverse reactions to the use of modeling substances in Cali, Colombia. A cross-sectional and descriptive study was used to determine the frequency of bacterial and fungal infections associated with complications from and adverse reactions to the use of modeling substances in 113 patients. We identified microorganisms in 22 patients and a frequency of 68.1% monomicrobial infections and 31.8% polymicrobial infections. The microorganisms identified in our study included *Bacillus cereus*, *Mycobacterium fortuitum*, and *Pseudomonas stutzeri*, among other microorganisms. The presence of adverse effects derived from the use of illegal modeling substances has been demonstrated; among these effects, infections occur with high frequency and place the health of the patient at risk and increase problems in health care.

## Introduction

Filler substances are injectable products that are used to modify anatomical appearance for aesthetic purposes and have been classified based on the time of absorption and biodegradable characteristics [1]. The infiltration of foreign materials not approved for medical use, such as paraffin, liquid silicone, acrylates, vitamins, vegetables or mineral oil among others, into soft tissue represents a serious health problem [2–4].

Examples of complications include aesthetic failure, ulceration, fistulae, infection, necrosis, inflammatory granulomatous and migration of filler material that can compromise vital organs and even cause death [1, 4]. Additionally, there are psychological, social, and labor impacts that carry an increased risk for morbidity and mortality. The complications can occur early (days to weeks) or late (weeks to years) after the procedure [5]. Filler substances can act as an adjuvant or a foreign substance, stimulating the immune response, and have the potential to induce an inappropriate autoimmune response known as autoimmune/inflammatory syndrome induced by adjuvants (ASIA) [6].

Infections can occur at any time after the procedure, and their occurrence is closely related to the aseptic conditions and the technique used by the staff during the process of infiltration as well as fluctuations of the immune system and the transient microbiota present on the hands of health personnel who do not use personal protective equipment [7]. The breakdown of skin integrity, the number of skin perforations, and the type of substance are associated with the development of skin infections by filler substances [8, 9].

In seeking to improve physical appearance, nonsurgical aesthetic procedures have appeared that involve the injection of filler modeling substances not approved for medical use under the premise that are simple methods, minimal invasiveness, safety, painlessness, immediate results, anonymity, and lower economic costs [10]. The infiltration of nonmedical filler substances for aesthetic procedures is a documented clandestine practice in several places around the world, including Colombia [3, 11]. The number of patients undergoing injectable soft-tissue fillers is not publicly available because most of these procedures are performed in nonlicensed facilities with unauthorized materials by unqualified personnel [6, 11]. In Colombia, it is estimated that more than 35,000 people have undergone some type of surgical procedure with biopolymers, of which more than 50% have some complications [7].

Widespread use of nonmedical fillers for cosmetic purposes has led to an increase in the reported number of late adverse reactions following injection and represents a serious health problem. The aim of this study was to identify microbial infections in patients with adverse reactions to the use of modeling substances in Cali, Colombia.

## Methodology

A cross-sectional descriptive study was conducted between January 2020 and April 2021 to determine the frequency of bacterial and fungal infections associated with complications and adverse reactions to the use of modeling substances in 113 patients who consulted with a clinic specializing in reconstructive aesthetic surgery and the removal of biopolymers (Santuario Medical Center) in the city of Cali, Colombia.

Briefly, in the biopolymer removal surgical procedure, the incisions were made along the demarcated lines in the form of “Angel wings”, which depends on the location of the granulomas and deep tissues affected identified by magnetic resonance imaging. Plane dissection was performed up to the muscular aponeurosis and subsequent block resections. Lower back, upper gluteal region (⅔), hips, and muscle dissections were performed. Dead space closure, planar synthesis, drain placement, and vacuum-assisted closure (VAC) therapy. With this technique, it is possible to eliminate the vast majority of biopolymers and diseased tissue, not only from the buttocks but also from those areas where the substance frequently migrates: the lower back, hips, and legs. One hour before the procedure, the patients received prophylactic antibiotic therapy with vancomycin and aztreonam and after surgery, antibiotic therapy was continued orally (cefadroxil) for 7 days. The patients were monitored periodically for six months. Most of the patients had improvement in local and systemic symptoms, as well as in their quality of life.

To collect patient information, a survey was used to gather data on demographics and the context of the application of the modeling substance. Clinical data were obtained from the medical consultation and the patient’s clinical history. Each survey was assigned a unique registration number.

### Ethical considerations

The study was conducted in accordance with the Declaration of Helsinki [12] and was approved by the Ethics and Research Methodology Committee of the National Institute of Health (Code CTIN-21-2017). Written signed consent was obtained from all the patients.

### Samples

During surgery, five tissue samples were collected from the area from which the modeling substance was removed, placed in Falcon tubes with 5 ml of culture broth or sterile saline solution, held at 4°C and sent to the Microbiology Laboratory of the National Heath Institute-Colombia within 24 h to be processed according to standard microbiological culture procedures.

#### Identification of aerobic and anaerobic microorganisms

For aerobic microorganisms, the tissue samples were collected in brain heart infusion (BHI) broth and incubated at 37°C for 24 h. For anaerobic microorganisms, samples were collected in thioglycolate culture broth and incubated at 37°C under anaerobic conditions. Aliquots of 50 μl of culture broth were plated in BHI, MacConkey, and sheep blood agar and incubated at 37°C for 24 to 72 h.

#### Identification of fungal structures

For the identification of fungal colonies, the samples in sterile saline solution were cut into small pieces with a sterile scalpel in a petri dish and inoculated directly onto Sabouraud agar medium, using a slight pressure. The media were incubated at 37°C and inspected every 24 h for 7 d. All recovered isolates were stored in 20% semiskimmed milk at -70°C in the INS strain of the Microbiology Group.

#### Identification of isolates by MALDI-TOF mass spectrophotometry

From a bacterial or fungal colony (CFU), an analysis of the protein profile of the microorganism was performed using MALDI-TOF mass spectrophotometry with Biotyper Bruker MALDI version 2.0 (Bruker Daltonics, Billerica, MA) containing the CE IVD and Bruker Daltonics databases.

#### Identification of mycobacteria

The tissue samples collected in sterile saline solution were homogenized with a vortex mixer for two minutes and centrifuged twice at 4000 × g for 5 min and 30 min. Two hundred microliters of the sediment was transferred to two plates with Löwenstein-Jensen culture media; one plate was incubated at 37°C and one was incubated at room temperature for up to 6 weeks before confirming a negative result.

#### Molecular study of samples

An analysis of the 16S rRNA gene sequence was used to identify bacteria from a tissue sample collected in sterile saline. DNA extraction was performed using the DNeasy® Blood & Tissue nucleic acid extraction kit (Qiagen). The genetic material obtained was used to amplify the 16S rRNA gene [13] using the human RNAseP gene as a PCR internal amplification control. The amplification products of the 16S rRNA gene were sequenced in the forward and reverse sense (Macrogen, South Korea). The sequences were visualized and edited using the DNA Baser Assembler and the Bio Edit Sequence Alignment Editor software (v. 7.2), exported in FASTA format and assigned a taxonomic classification by alignment using the nucleotide Basic Local Alignment Search Tool (BLASTN) against the nucleotide collection database (NCBI database). Additionally, samples that amplified the 16S rRNA gene were processed for classification into gram-positive or gram-negative samples using real-time PCR according to the method of Wu Y-D. *et al*. [14].

For mycobacteria isolates, DNA extraction was then performed as described by Van Soolingen *et al*. [15]. For the genotypic identification of mycobacteria using the methodology of PCR-restriction analysis (PRA), the protocol described by Telenti was followed [16]. Digestion results were compared using the Prasite database http://app.chuv.ch/prasite.

Based on the results obtained, the recovered isolates were subjected to different phenotypic and genotypic characterization tests, including antimicrobial susceptibility testing [17], serotyping using the Kauffmann-White Salmonella scheme, pulse-field-gel electrophoresis (PFGE) of *Bacillus cereus* with the *Not*I macrorestriction enzyme according to the procedure previously described [18] and whole-genome sequencing on the Illumina MiSeq platform (Illumina, San Diego, CA, USA). The assembled genomes were analyzed with the BTyper tool (version 2.3.2) and PATRIC workspace (https://www.patricbrc.org/) for the identification of MLST profiles, phylogenetic relationships, antimicrobial genes and virulence factors. The draft genomes of *B*. *cereus* have been deposited in GenBank as BioProject PRJNA849146.

### Data analysis

Patient data were recorded in an Excel database for analysis. Continuous variables were reported as measures of central tendency, and count data were reported as absolute frequencies and presented as percentages. Pathogenic microorganisms were considered only when they were present in two or more tissue samples identified by culture or 16S rRNA PCR. Contamination was considered possible when a microorganism was isolated in only one sample. Polymicrobial infection was defined as the isolation of more than one other microorganism, excluding contamination.

## Results

### Demographic and clinical characteristics

**Fig 1:**
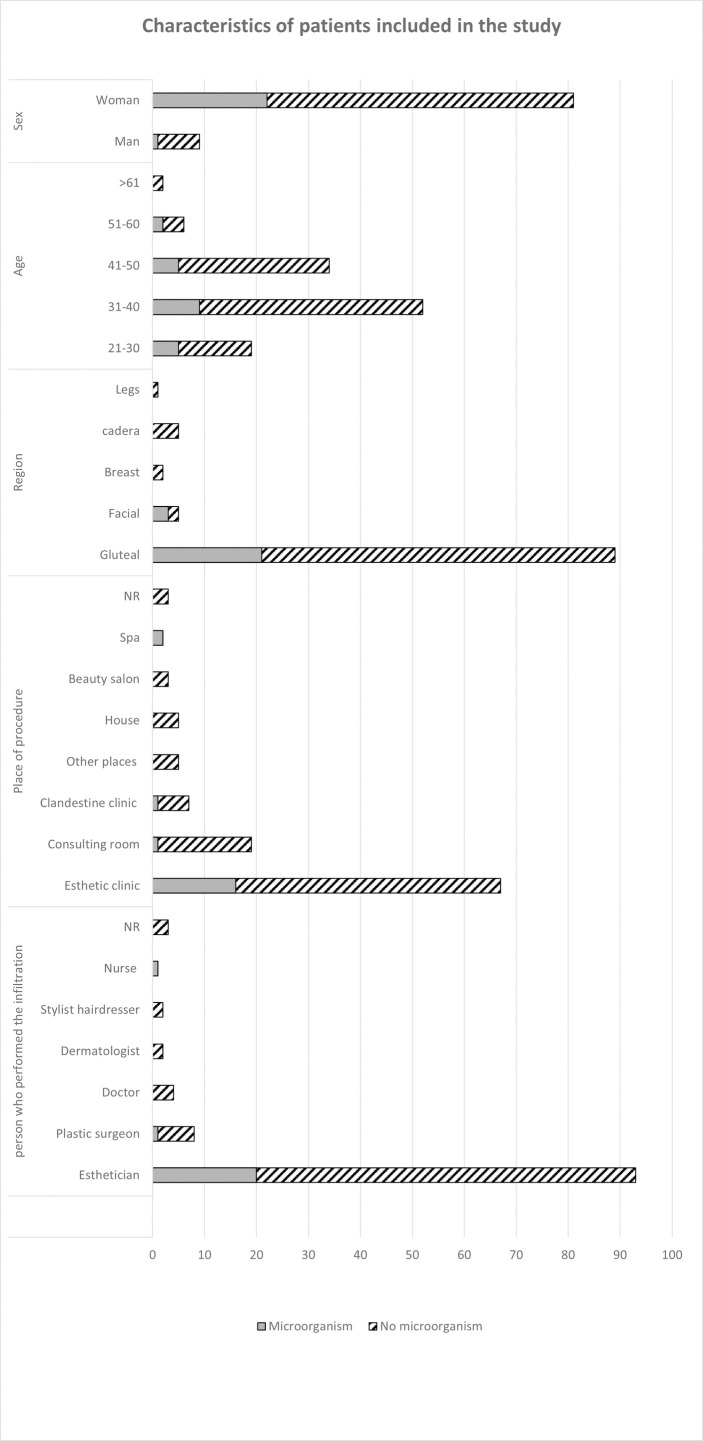
Characteristics of patients included in the study.

Of the 113 patients, 103 (91.2%) were female, and 78 (69.0%) were from Colombia, with an average age of 37 years (range, 21 to 64 years); however, 40% of the patients were between 32 and 41 years old at the date of diagnosis. The most affected anatomical area was the gluteal region with 110 (97%) patients, followed by the facial region with eight cases (7%). Eleven (9.7%) patients had more than one infiltration area (Fig 1).

The average time from injection of the modeling substances was 7.8 years (range, 1 to 11 years), and the time to presentation of signs and symptoms from the injection of the substance was 4 years (range, 1 to 10 years). The most representative clinical manifestations observed in medical consultation were asymmetry 107 (94.7%) and a granulomatous reaction 106 (93.8%).

The procedure had been performed mainly in aesthetic clinics (n = 67, 59.3%) and medical consulting rooms (n = 19, 16.8%) by estheticians (n = 91, 80.5%) and to a lesser extent by medical staff (n = 16, 14.1%) (Fig 1). Most of the patients had no knowledge of the infiltrated substance (n = 80, 70.7%), but ten (8.8%) and five (4.4%) patients reported Biogel and hyaluronic acid, respectively, as the infiltrated substance.

### Microbiological analysis

Based on the phenotypic and molecular results, at least one microorganism was identified in 22 (19.5%) patients, and infections with more than one microorganism were detected in seven (6.2%) patients (Table 1).

**Table 1:** Microbiological identification by culture and molecular tests in tissue samples from patients.

Patient code	Bacterial strains identified	Conventional PCR from tissue		16S rRNA sequencing
		RNasa P gene	16S rRNA gene	
3	*Stenotrophomona maltophilia*	+	+	ND
	*Bacillus cereus*			
	*Lysinibacillus boronitolerans*			
5	*Staphylococcus warneri*	+	+	ND
	*Bacillus oceanisediminis/ Bacillus firmus*			
	*Pseudomonas stutzeri*			
9	*Bacillus cereus*	+	+	*Bacillus cereus*
12	*Bacillus cereus*	+	+	ND
	*Pseudomonas stutzeri*			
13	*Pseudomonas stutzeri*	+	+	ND
	*Bacillus cereus*			
14	Without isolation	+	+	*Mycobacterium sp*
18	*Mycobacterium fortuitum*	+	+	ND
19	*Mycobacterium fortuitum*	+	+	ND
25	*Bacillus oceanisediminis*	+	+	*Bacillus sp*.
28	Without bacterial isolate	+	+	*Pseudomonas sp*.
39	*Enterococcus faecalis*	+	+	ND
	*Staphylococcus epidermidis*			
41	*Bacillus cereus*	+	+	*Bacillus cereus*
42	*Bacillus cereus*	+	+	ND
	*Mycobacterium fortuitum*			
45	*Bacillus cereus*	+	+	*Bacillus cereus*
51	*Candida parapsilosis*	+	+	ND
52	*Cutibacterium avidum*	+	+	ND
63	*Mycobacterium fortuitum*	+	+	ND
	*Staphylococcus capitis*			
77	*Staphylococcus hominis*	+	+	*Staphylococcus sp*.
96	*Staphylococcus lugdunensis*	+	+	*Staphylococcus sp*.
107	*Mycobacterium fortuitum*	+	+	ND
115	*Salmonella sp*	+	+	* *ND
116	*Salmonella sp*	+	+	* *ND

*(+): Gene amplification, (-): No gene amplification, (ND): Not determined.

In two patients, identification of bacteria at the genus level was conducted by sequencing the 16S rRNA gene. All isolates were recovered from the gluteal region of patients who had pain, local temperature increase, erythema, deformity, and inguinal lymphadenopathy. Only seven patients reported having knowledge of the infiltrated substance.

A total of 24 isolates were identified by MALDI-TOF to the species level with scores ≥ 2.0 (S1 File), and two bacterial genera were identified by 16S rRNA sequencing (Table 1). The most frequently isolated microorganisms were *Bacillus cereus* (n = 7, 31. 8%) and *Pseudomonas stutzeri* (n = 3, 13. 6%). Five different species of *Staphylococcus* were identified. *B*. *cereus* and *P*. *stutzeri* were predominantly found in polymicrobial infections and were recovered in four patients. Two microorganisms were identified by sequencing.

**Fig 2:**
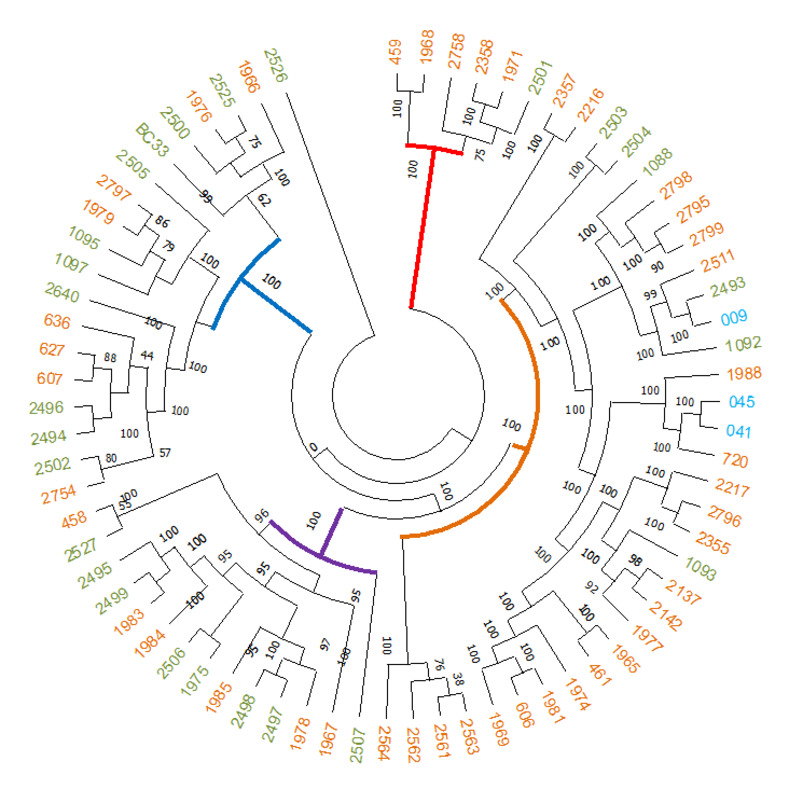
Phylogenetic relationships of *B*. *cereus* isolates sequenced with pathogenic and non-pathogenic strains sequences. In blue color isolates recovered in this study, in orange and green colors non-pathogenic strains and pathogenic strains, respectively.

Given that *B*. *cereus* is a ubiquitous bacterium and an opportunistic human pathogen, we performed the genetic characterization of strains isolated by culture from tissue samples obtained during the removal of biopolymers. Two PFGE patterns (A and B) were identified; pattern A clustered four isolates with identical band patterns, and pattern B clustered only one isolate. Two isolates PFGE A and the B isolate were selected for WGS for further analysis of virulence factors and associated with pathogenic and nonpathogenic strains. Based on the bioinformatics tools used, 72 genes were identified, of which 30 were associated with virulence factors, 33 with biofilm development and nine with resistance genes, according to reports by Bianco *et al*. [19] (S2 File). The phylogenetic relationships between isolates sequenced in this study with pathogenic (n = 53) and nonpathogenic (n = 27) strains revealed four phylogenetic clades (Fig 2) (S3 File). The major clade (n = 33 isolates) consisted of mainly pathogenic strains (n = 25) and included isolates 09, 041, and 045 from this study. The other clades were mainly associated with environmental strains.

*Mycobacterium* isolates were identified in 4 patients with monomicrobial infections and in 2 patients with polymicrobial infections. Using amplification of the *Hsp*65 gene and restriction endonucleases for the identification of nontuberculous rapid growers (rate of growth: 14 days), the isolates were classified as *Mycobacterium fortuitum*, and one isolate was not an established species. *Pseudomonas stutzeri* was identified in three patients, and the susceptibility test showed only one isolate with resistance to ceftazidime, cefotaxime, ceftriaxone and meropenem. Two isolates of *Salmonella* serovar Enteritidis with pansusceptible phenotypes were recovered. *Candida parapsilopsis* was identified by MALDI-TOF in a tissue sample.

## Discussion

The injection of certain filler substances for cosmetic purposes is illegal throughout the world; however, it is a common practice in many countries and regions, such as Latin America and Asia, and has reached epidemic proportions [11]. This is a study of 113 patients who developed adverse reactions after exposure to biopolymers. The majority of patients were Colombian women, but some patients were of other nationalities. Esthetic augmentation of the gluteal region was the main procedure performed by nonmedical professionals or unqualified persons under nonaseptic conditions. In 22 patients, bacterial or fungal isolates were identified from samples obtained during surgery for the removal of biopolymers.

The most affected area in our patients was the buttocks, which is consistent with gluteal augmentation being one of the most common procedures for achieving improvement in the gluteal contour [2, 20, 21]. The procedures were conducted at unauthorized and clandestine sites with filler substances that have not been approved for aesthetic purposes. Regulations that prevent the use of unknown filler substances, monitor the personnel who use filler substances, and warn people about the dangers of these procedures are necessary [4].

In our patients, the average time interval between the injection of modeling substances and the presentation of clinical manifestations was 4 years. Complications with permanent fillers may occur years after implantation [22], and it has been determined that one of the main manifestations of the injection of filler substances is a chronic local inflammatory reaction accompanied by multiple symptoms and signs, with the onset of symptoms at 4.3–4.5 years [2].

An effective treatment has not been well established. Most of the time, it requires a multidisciplinary approach that should include surgical procedures, systemic therapy and mental health care. Several surgical techniques have been proposed which include extensive surgical resection and subsequent reconstruction, but there is no standard treatment and the scientific literature is scarce. However, surgical removal can be difficult given that large amounts of injected modeling agents tend to mix with healthy tissue. Other techniques included conventional liposuction and ultrasound-assisted liposuction [6, 11, 23].

Injectable fillers are also associated with microbial infections. We identified 31 microorganisms from tissue samples of patients with injected modeling substances. It has been established that after cosmetic dermal filler injections, bacteria of low virulence can produce infections several months after the procedure, and these are associated with the formation of biofilms or nonattached aggregated bacterial cells [24]. Because the identification of biofilms is difficult, culture tests are typically negative, and complications are attributed to the immune response to filler substances [22, 25]. The inflammatory process may involve an active granuloma years after injection, which may be related to the development of bacterial biofilms or microbial colonies encapsulated in the extracellular matrix surrounding the foreign body, leading to chronic nonsymptomatic infections with gradual reactivation [26–28]. Infectious agents can be introduced through direct inoculation during the initial injection of filler substance, and the filler can serve as a focus for microbial infection or there can be hematologic spread of a systemic infection [25, 28–30]. Adverse reactions to tissue fillers used for esthetic purposes have been associated with reactions caused by an autoimmune response to the injected gel filler, but studies have revealed that these reactions can be caused by bacterial infection [31–34].

Our study showed a frequency of 68.2% monomicrobial infections and 31.8% polymicrobial infections. Polymicrobial infections suggest the development of microbial biofilms composed of microbial communities in which bacterial strains that are not pathogenic under normal circumstances may act as pathogens when they are associated with foreign bodies [32, 33]. Crowe et al. suggested that biofilms are more diverse than those found in previous studies by culture and may be heterogeneous and composed of a diversity of relatively abundant species.

*Bacillus cereus* was the main microorganism recovered from tissue samples during the surgical extraction of the modeling substance. *B*. *cereus* remains an important cause of enteric infections, skin infections and systemic infections [31, 34, 35]. Subclinical bacterial infections can be caused by the formation of biofilms on biomedical devices, and *B*. *cereus* strains are able to persist and remain a source of infection, most likely due to their ability to form biofilms [15, 36–38]. WGS of a subset of the isolates studied showed characteristics of pathogenic isolates based on the identification of virulence-associated genes *(NHE*, *entA*, *entFM*, *sph*, *cerA*, *cerB*, *inhA*, *plcA*, and *plcB*), biofilm formation genes (*PlcR*, *calY*, *tasA*, *tapA*, *sipW*, and *CodY*) and the presence of several genetic mechanisms for resistance to beta-lactam, vancomycin, and fosfomycin antibiotics, according to previous reports on human clinical strains from blood cultures from hospitalized patients [19, 35]. The *B*. *cereus* genome sequences of isolates obtained in this study were compared with nonpathogenic and pathogenic *B*. *cereus* strains. Interestingly, isolates from patients with adverse reactions to fillers tended to integrate with a cluster that is phylogenetically related to the group of pathogenic strains. This result suggests the ability of *B*. *cereus* to cause infection, and it should be considered a risk factor in patients who use illegal modeling substances.

We identified six *Mycobacterium* sp., of which five corresponded to the genus *M*. *fortuitum*. Non-tuberculous mycobacterial (NTM) infections have been associated with the infiltration of filler substances by direct inoculation, contiguous or hematogenous dissemination and the use of contaminated substances and are favored by their ability to survive in a variety of environments, such as water and soil, allowing their adherence and resistance [39–43]. *Mycobacterium fortuitum*, *M*. *chelonae* and M. *abcessus* are listed as the most frequent species causing skin and soft tissue infection [43]. *M*. *fortuitum* has been reported most frequently as an infectious agent in cosmetic treatments by direct inoculation or an invasive procedure, unlike other NTMs associated with cutaneous involvement, which have been isolated after other cosmetic procedures such as lipolysis, face rejuvenation, mesotherapy, liposuction, and liposculpture of the breasts and buttocks [41, 42, 44]. *M*. *fortuitum* tends to present with a single lesion as an erythematous papule that varies in size and may develop into boils or inguinal adenopathy, occasionally causing infections at the surgical site; they manifest late, and they grow in biofilms [45–47]. Consequently, it is necessary that physicians caring for such patients request acid-fast bacilli staining and mycobacterial culture for the diagnosis of mycobacterial infections in the skin and soft tissue of patients with aesthetic procedures [42].

*Pseudomonas* sp. is considered an opportunistic bacterial pathogen. *P*. *aeruginosa* has been implicated as a cause of both acute and late-onset infections in cosmetic soft tissue augmentation [48, 49]. To date, *P*. *stutzeri* infection has been reported in bacteremia, pneumonia, osteomyelitis, arthritis, and endocarditis [50, 52]. However, most cases have been documented in patients with risk factors such as immunosuppression, the presence of comorbidities, a previous history of surgery, trauma or skin infections and are associated with the use of foreign bodies as bioprosthesis devices [50–52]. In our study, the possible source of infection with P. *stutzeri* in patients with modeling substances may be the result of contamination during the cosmetic procedure or bacteria residing within the filler substance, where they can persist for years.

Microorganisms that are considered integral parts of the cutaneous microbiota, such as *Sthaphylococcus sp*. and *Cutibacterium sp*., were isolated at lower frequencies. Commensal bacteria of low virulence are capable of acting as opportunistic pathogens, producing long-term infection in patients who use filler substances in cosmetic procedures, possibly due to biofilm formation [10, 25, 53, 54].

This study has limitations. 1) The data are not population-based, and all cases were referred and treated in a private clinic specializing in reconstructive aesthetic surgery and the removal of biopolymers. Other studies should be conducted in cities where the same problem occurs. 2) During the development of the study, no environmental samples were taken; however, the surgery rooms of the clinic are certified and conform to quality parameters established by the National Ministry of Health. 3) Because the patients could not identify the filler substance, no associations could be made between the modeling substance and the identified microorganisms. 4) Another limitation was the inability to demonstrate bacterial biofilm formation in clinical samples from patients and assess the biofilm formation capacity of isolated bacteria.

## Conclusions

Procedures that involve injectable nonmedical materials as filler modeling substances for aesthetic purposes can expose the patient to the development of infectious processes. The possible source of late infections in these patients may be the result of environmental contamination at the time of the cosmetic procedure or the use of potentially contaminated filler material. It is important to recognize the danger of such illegal procedures and to increase the awareness of the public to minimize these adverse events, as this has evolved into a significant public health issue.

Of course, the risk of infection with non-medical substances is higher, but this does not emerge specifically from the available data set.

## Supporting information

S1 File Table MALDI_TOF data. (XLSX)

S2 File Table genes identified. (XLSX)

S3 File Table strains. (XLSX)
